# Getting out of a wheelchair: an uncommon insertion mutation in exon 19 of EGFR responsive to erlotinib

**DOI:** 10.1186/2193-1801-3-507

**Published:** 2014-09-09

**Authors:** Abed Agbarya, Meira Melamed-Frank, Orit Kaidar-Person, Ilana Goldberg-Cohen, Haitam Nasrallah, Mira Wollner, Jair Bar, Gad Rennert, Flavio Lejbkowicz

**Affiliations:** Division of Oncology, Rambam Health Care Campus, POB 9602, Haifa, 31096 Israel; Carmel Medical Center and Bruce Rappaport Faculty of Medicine, Technion-Israel Institute of Technology, Haifa, Israel; Oncology Institute, Tel Hashomer Medical Center, Tel Aviv, Israel

**Keywords:** Epidermal growth factor receptor, Erlotinib, Exon 19 insertion, Non-small cell lung cancer

## Abstract

**Background:**

Most patients with non-small cell lung cancer (NSCLC) present with advanced disease and have poor long-term prognosis. Advanced NSCLC that contains characteristic mutations in epidermal growth factor receptor (EGFR) are highly sensitive to EGFR tyrosine kinase inhibitors (TKIs). EGFR exon 19 insertions mutations are rare, and response to TKIs is still unclear.

**Case description:**

A young Arab patient was diagnosed with metastatic disease of NSCLC harboring an exon 19 insertion of 18 nucleotides. The patient showed a very impressive clinical and radiological response within few weeks treatment with TKI agent.

**Discussion and evaluation:**

To our best knowledge, This case is the first case in Arab woman and one of few cases described in the literature with this rare mutation responding to TKIs.

**Conclusions:**

Treatment with TKIs should be the standard choice in patients with metastatic disease NSCLC.

## Introduction

Most patients with non-small-cell lung cancer (NSCLC) present with advanced disease and have a poor long-term prognosis. Advanced NSCLC that contains characteristic mutations in EGFR (epidermal growth factor receptor) are highly sensitive to EGFR tyrosine kinase inhibitors (TKIs), such as gefitinib or erlotinib, and analysis for the presence of a driver mutation in EGFR is the standard approach in the initial workup of a patient with advanced NSCLC. These mutations are most frequently observed in adenocarcinomas, females, non-smokers, and the Asian population [Chan et al. 2013; Mok et al. 2009]. As previously documented [Mok et al. 2009], EGFR exons 18, 19, and 21 are the mutation-sensitive regions rendering a positive outcome in TKI therapy with response rates and progression free survival (PFS), up to 70% and 13 months, respectively . Exon 19 deletions of 15–18 bp represent more than 50% of the mutations in EGFR, and exon 21 point mutation at the residue L858R represents more than 30%. Patients harboring one of these mutations have a relatively good outcome with TKI treatment.

EGFR exon 19 insertions mutations are not commonly reported, and no more than 20 cases have been described to date [He et al. 2012]. Interestingly, all these cases presented some similarities. Mostly, patients are female, non-smokers, harboring an 18 nucleotides insertion. Therefore, this insertion results in an additional six amino-acids. The outcomes for treatment with (TKIs), in this type oif mutation is not known since only few patients received such treatment [He et al. 2012].

We describe for the first time the case of a young Arab woman harboring an exon 19 insertion of 18 nucleotides who showed a positive outcome after three months of treatment with TKI.

## Method

EGFR mutations are identified from tumor specimens from patients with NSCLC using DNA sequencing, RT-PCR or fragment length analysis. Briefly, DNA was extracted from paraffin-embedded tumor samples using a commercially available kit, according to the manufacturer’s recommendation (QIAmp DNA mini kit, Qiagen). Genotyping of exons 18, 20, and 21 using SNP Assay-by-Design was performed by allelic discrimination using a Taqman- based SNP genotyping assay on the ABI Prism 7900HT Sequence Detection System (Applied Biosystems, Foster City CA, USA). The assay was performed in a 20 μl reaction volume containing 1 μl genomic DNA, 0.15 μl primer/probe mix, 5 μl TaqMan genotyping master mix (Applied Biosystems), and 14 μl of double distilled water. The thermocycling set-up includes a pre-run of 2 minutes at 50°C, followed by 10 minutes at 95°C; then 50 cycles with 10 seconds at 95°C, followed by 60 seconds at 60°C. Primers and probes were generated by the Assay-by-Design custom oligonucleotide reagent service (Applied Biosystems) and are available upon request. In parallel and independently, all samples were sequenced for exons 18, 19, 20, and 21. Direct sequencing reactions were performed in the ABI 3130 Sequencer. Fragment length analysis isolates the EGFR exon 19 region (a fragment spanning amino acids 700–800) via PCR reaction with the following FAM labeled primers:

Forward 5′ - FAM -GTGCATCGCTGGTAACATCC -3′,

Reverse 5′ -TGTGGAGATGAGCAGGGTCT – 3′.

PCR products were diluted 1:10 and 1 μl was added to a reaction solution containing 8.5 μl Formamide and 0.5 μl GeneScanTM – 500 ROX™ Size Standard (Applied Biosystems). Fragment analysis was performed with the 3130xl Genetic Analyzer (Applied Biosystems). Deletions and/or insertions were clearly observed by a change in the fragment size.

## Case description

A healthy, non-smoking, 39-year old female of Arabic origin was diagnosed with Stage IV adenosquamous cell carcinoma of the lung. The patient had no family history of malignancy. In the four months prior to the diagnosis of lung cancer, she suffered from dyspnea, cough, and recurrent episodes of pneumonia, despite antibiotic treatment. CT scan of the chest revealed massive left pleural effusion, a mass in the left lower lobe, and a nodule in the left upper lobe. Cytology from the pleural effusion was suspicious of malignancy. Biopsy taken during bronchoscopy revealed adenosquamous cell carcinoma. Immunohistochemistry was positive for cytokeratin 7, partially for p63, and negative for cytokeratin 20, TTF1, ER, WT1, mammoglobin, and GCDFP 15. PET-CT showed pathological FDG uptake in the left lower lobe, left lung hilum, mediastinal lymph nodes, left pleura, and lytic bone lesions.

The patient was admitted to hospital due to severe dyspnea requiring an oxygen mask, confined to a wheelchair due to fatigue and dyspnea, and suffered uncontrollable pain mandating opioid treatment. She was found to have an Eastern Cooperative Oncology Group (ECOG) score performance status of 3.

**Figure 1:**
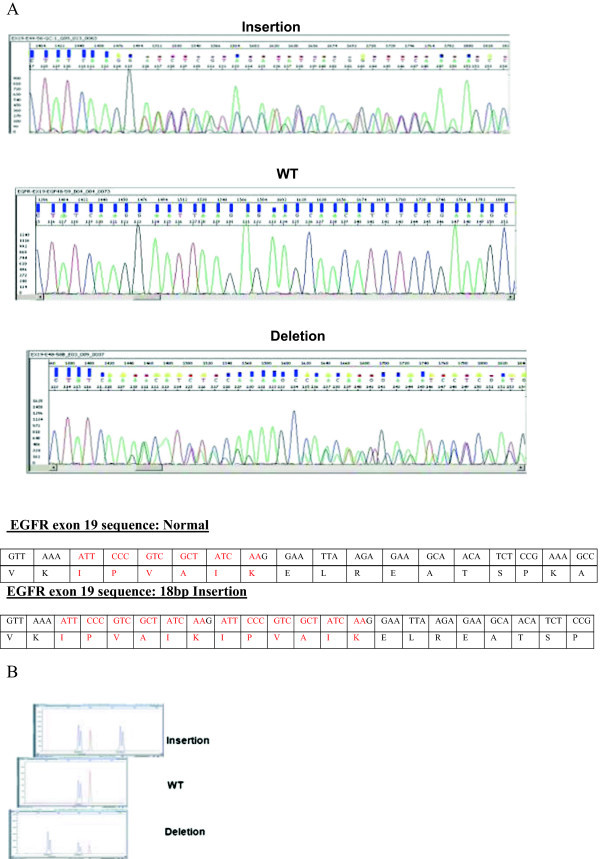
**Molecular analysis of the EGFR exon 19 insertion. A)** Direct sequencing: Sanger sequence was performed and detected the mutation in the EGFR exon 19. Below are the electropherograms of insertion, normal (WT) and deletion sequences. The description of the normal and insertions sequences are described with their relative amino-acid. **B)** Fragment length analysis of EGFR exon 19: A very precise and simple visual way to detect and describe the insertion of 18 nucleotides in the EGFR exon 19. The picture shows a comparison between the insertion with the wild type and a deletion of EGFR exon 19.

A sample of the patient’s biopsy was examined for all EGFR mutations. In the fragment length analysis assay of exon 19, there appeared an uncommon pattern of an insertion of 18 nucleotides. The EGFR exon 19 sequence was deciphered by direct sequencing. We found that the sequence c.2237ins ATTCCCGTCGCTATCAAG was repeated as demonstrated in Figure 1, showing the 18 bp insertion and resulting in a substitution in the protein (p.K745_E746insIPVAIK).

**Figure 2:**
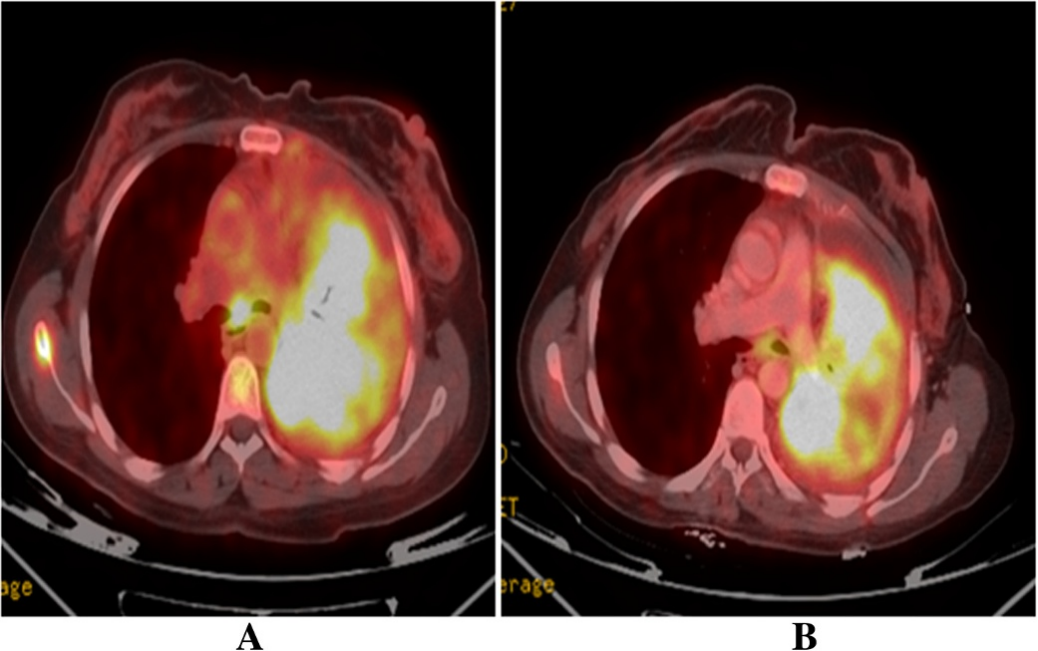
**PET-CT scan before (A) and after (B) three months of treatment with erlotinib, showing improvement in the mediastinal lymph node and left lung uptake.**

After reviewing the published data, which included a report of an uncommon insertion mutation in exon 19 responsive to tyrosine kinase inhibitors [Chan et al. 2013], the patient was started on erlotinib 150 mg daily. Rapid symptomatic improvement followed and, at the one-month follow-up visit, her performance status was evaluated as ECOG 0, not requiring any more oxygen or opioids. PET-CT scan after three months of treatment with erlotinib showed an improvement in all disease sites, including the bone lesions (Figure 2). A good partial response was documented in all the sites of the disease including the left lung mas, mediastinal lymph nodes and the lytic lesions in the bones. The patient responded to erlotinib for nine months until symptomatic and radiological progression. She is now receiving a combination chemotherapy with Cisplatin, Pemetrexed and Bevacezumab, for two months, with a clinical improvement.

## Discussion

Several studies have demonstrated that TKIs render a favorable outcome when administered as treatment for pulmonary adenocarcinoma in the presence of a somatic EGFR mutation [Mok et al. 2009]. EGFR exon 19 insertion is an uncommon mutation and poorly described [He et al. 2012]. In the analysis of this specific case, we found a duplication of 18 nucleotides in exon 19 leading to an insertion of the amino-acids IPVAIK. According to molecular modeling, this insertion in the EGFR gene modifies the kinase domain and may facilitate the binding of TKIs [Otto et al. 2012], leading to sensitivity to these agents. In addition, the P747L (substitution of a proline for leucine), a rare mutation at the border of the insertion described in this case, seems to be critical for both exon 19 deletions and insertions [He et al. 2012]. He et al. 2012 describe 12 cases harboring exon 19 insertions. Their patients are mostly females who never smoked, as was our patient.

In the last few years, with the evolution of molecular medicine, there is a paradigm shift in the treatment of patients with NSCLC who present with advanced disease. The presence of EGFR-activating mutations in patients with lung adenocarcinoma was found to be associated with sensitivity to TKIs, such as gefitinib or erlotinib [Mok et al. 2009]. Approximately 90% of these mutations are exon 19 deletions or exon 21 L858R point mutations [Ladanyi and Pao 2008]. Other EGFR mutations, such as exon 20 insertion, have been associated with much lower response rates to TKIs [Mok et al. 2009]. This case of exon 19 insertion is unique, as histology indicated adenosquamous cell carcinoma of the lung which is not commonly associated with EGFR mutation [Jänne and Johnson 2006] and uncertain data about the sensitivity of this mutation to TKIs. The clinical scenario is also unique, as this is the first known case of a patient of Arabic descent to have EGFR mutation. Only a few small reports have dealt with the prevalence of EGFR mutation in Arab patients [Errihani et al. 2013]. According to the Israeli Ministry of Health Cancer Statistics, lung cancer is common in Arab males (1/12) due to a high rate of smoking, and the prevalence of lung carcinoma has increased in the past decade in Arab females (1/70), but is still less frequent than in females of Jewish descent (1/45) (NCCN Clinical Practice Guidelines IN Oncology (NCCN Guidelines) 2014).

Although the patient presented in this case had significant symptoms, we decided to initiate first-line treatment with TKI over conventional chemotherapy. Takeda et al. [2014] indicated that some patients experience rapid tumor regression (≤4.2 weeks) or a high degree of tumor shrinkage (≥56%), whereas platinum combinations generate an overall response rate of approximately 25-35%. Moreover, patients in poor condition of ECOG 3–4 do not generally benefit from cytotoxic treatment, with TKIs for EGFR mutation-positive patients forming an exception to this rule [Jänne and Johnson 2006].

Our patient improved rapidly, without any significant toxicity from erlotinib, similar to that described by others [Chan et al. 2013; Takeda et al. 2014]. However, it is uncertain whether other uncommon EGFR mutations are associated with sensitivity to EGFR TKIs. Previous reports and trials indicate that uncommon EGFR mutations (such as G719X or L861Q) are associated with a shorter response to TKIs [Chan et al. 2013; Lee et al. 2013; Takeda et al. 2014; Wu et al. 2007]. A multi-institutional effort is needed to combine the data available, in order to predict the quality of TKI treatment in these cases.

## Conclusions

To the best of our knowledge, we describe for the first time a case of a young Arabic woman harboring an exon 19 insertion of 18 nucleotides who showed a positive outcome after three months of treatment with TKI. This finding may contribute to the small number of reports of exon 19 insertion and further emphasize the therapeutic importance of TKI treatment. Our results seem to indicate that sequencing and fragment length analysis of EGFR exon 19 should be included on a routine basis, instead of relying on tests based on point mutations only. The use of two different methods to map the exon 19 insertion mutation may be crucial to ensure detection of exon 19 insertions.

### Consent

Written informed consent was obtained from the patient for publication of this case report and any accompanying images.
